# Variability of corticospinal and spinal reflex excitability for the ankle dorsiflexor tibialis anterior across repeated measurements in people with and without incomplete spinal cord injury

**DOI:** 10.1007/s00221-024-06777-z

**Published:** 2024-01-25

**Authors:** J. A. Brangaccio, A. M. Phipps, D. E. Gemoets, J. M. Sniffen, Aiko K. Thompson

**Affiliations:** 1 National Center for Adaptive Neurotechnologies and Stratton VA Medical Center, Albany, NY USA; 2 Department of Health Sciences and Research, College of Health Professions, Medical University of South Carolina, 77 President Street, MSC 700, Charleston, SC 29425 USA; 3 State University of New York at Stony Brook, Stony Brook, NY USA

**Keywords:** Diurnal rhythm, H-reflex, Spinal cord injury, Tibialis anterior, Motor evoked potential

## Abstract

To adequately evaluate the corticospinal and spinal plasticity in health and disease, it is essential to understand whether and to what extent the corticospinal and spinal responses fluctuate systematically across multiple measurements. Thus, in this study, we examined the session-to-session variability of corticospinal excitability for the ankle dorsiflexor tibialis anterior (TA) in people with and without incomplete spinal cord injury (SCI). In neurologically normal participants, the following measures were obtained across 4 days at the same time of day (*N* = 13) or 4 sessions over a 12-h period (*N* = 9, at 8:00, 12:00, 16:00, and 20:00): maximum voluntary contraction (MVC), maximum M-wave and H-reflex (*M*_max_ and *H*_max_), motor evoked potential (MEP) amplitude, and silent period (SP) after MEP. In participants with chronic incomplete SCI (*N* = 17), the same measures were obtained across 4 days. We found no clear diurnal variation in the spinal and corticospinal excitability of the TA in individuals with no known neurological conditions, and no systematic changes in any experimental measures of spinal and corticospinal excitability across four measurement days in individuals with or without SCI. Overall, mean deviations across four sessions remained in a range of 5–13% for all measures in participants with or without SCI. The study shows the limited extent of non-systematic session-to-session variability in the TA corticospinal excitability in individuals with and without chronic incomplete SCI, supporting the utility of corticospinal and spinal excitability measures in mechanistic investigation of neuromodulation interventions. The information provided through this study may serve as the reference in evaluating corticospinal plasticity across multiple experimental sessions.

## Introduction

Corticospinal activity is essential in human motor control (Ridding and Rothwell 1997; Capaday et al. 1999; Petersen et al. 2001; Pascual-Leone et al. 2002; Siebner and Rothwell 2003; Wirth et al. 2008a, 2008b; Barthelemy et al. 2010), and its plasticity is the key for learning motor skills and for re-learning them after central nervous system lesions (Classen et al. 1998; Karni et al. 1998; Butefisch et al. 2000; Liepert et al. 2000a, b; Rioult-Pedotti et al. 2000; Nudo 2003; Ziemann 2004; Ziemann et al. 2004, 2006; Ramanathan et al. 2006). To properly evaluate the corticospinal plasticity in health and disease, it is critical to establish whether and to what extent the corticospinal and spinal responses fluctuate systematically across multiple measurement times. In monkeys, rats, mice, and humans, the excitability of the soleus H-reflex pathway changes systematically throughout day (i.e., diurnal variation) (Wolpaw et al. 1984; Chen and Wolpaw 1994; Carp et al. 2006a; Lagerquist et al. 2006), but not across days (i.e., day-to-day variation) (Wolpaw 1987; Chen and Wolpaw 1995; Carp et al. 2006b; Thompson et al. 2009). In persons with no known neurological conditions, the soleus H-reflex and maximum voluntary contraction (MVC) force increase from the morning to evening, while the flexor carpi radialis (wrist flexor) H-reflex and MVC force do not change throughout day (Lagerquist et al. 2006), suggesting that diurnal variations in voluntary activation of the muscle and spinal reflex excitability may differ between muscles. Appearance of diurnal rhythm may also differ between the spinal reflex excitability and the corticospinal excitability, even in the same muscle; when the soleus corticospinal excitability was measured as the size of corticospinal motor evoked potential (MEP), its diurnal rhythm differed between participant chronotypes [i.e., morning people vs. evening people (Tamm et al. 2009)]. Thus, what emerges from the available studies is that the presence, pattern, and extent of systematic excitability variation likely differs between the pathways examined.

Currently, the presence of diurnal variation or the extent of the stability of the corticospinal excitability for the ankle dorsiflexor tibialis anterior (TA) is not known. Since the corticospinal drive is important for TA activation (Brouwer and Ashby 1992; Schubert et al. 1997, 1999; Capaday et al. 1999) and in particular during the swing phase of locomotion, enhancing the corticospinal drive for the TA could be an effective therapeutic strategy for augmenting rehabilitation of gait function in people after spinal cord injury (SCI) and other neuromuscular disorders (Thomas and Gorassini 2005; Everaert et al. 2010; Urbin et al. 2017; Thompson et al. 2019; Thompson and Sinkjaer 2020). To ensure the proper interpretation of clinical and mechanistic study findings involving TA corticospinal functions, this study set out to establish the variability and stability of TA corticospinal excitability in individuals with and without corticospinal lesions.

Specifically, in adults with no known neurological conditions, we examined diurnal (within-day) variability in the measures of spinal and corticospinal excitability for the TA and compared its extent and pattern with those of day-to-day variability. If, for instance, one of the measures showed a trend of increasing or decreasing in amplitude from the first (8:00) measurement to the last (20:00) measurement (i.e., diurnal variability is present), examining the variation trend across four days of measurements would allow us to see if such variation occurs when the measurements are simply repeated multiple times.

In adults with chronic incomplete SCI, we examined the extent of day-to-day variability in spinal and corticospinal excitability for the TA, across multiple days of measurements made at the same time of day. Note that, in the present study, we opted not to examine diurnal variability in participants with SCI. It would not be possible to control for the effects of medication that most of those individuals had been taking chronically daily; medication’s peak plasma levels, bioavailability, and pharmacological and physiological effects (Ghanavatian and Derian 2022) would not be the same throughout day, and medication effectiveness would also be hard to estimate or control for throughout day for each person. Thus, in individuals with SCI, we focused on estimating the day-to-day variability in various measures of spinal and corticospinal excitability.

## Methods

### Study participants

In the present study, to examine the variability in the corticospinal and spinal responses within and across days, measurements were made in two separate series of experiments. Of 16 individuals with no known neurological conditions who participated in the study, 9 (3 men and 6 women aged 36 ± 10 years) were studied with the within-day series of experiments and 13 (6 men and 7 women, aged 33 ± 7 years) were studied with the across-day series of experiments. Six individuals participated in both the within-day and across-day experiments. In this study, we set the minimum sample size of *N* = 9 for each experiment, assuming a 20% change in the H-reflex value as observed in Lagerquist et al. (2006). Over the four measurement time points, the sample size of *N* = 9 would detect a statistically significant systematic within-day or across-day change with 84% power.

Seventeen adults (13 men and 4 women, aged 50 ± 11 years) with well-defined stable impairment of weak ankle dorsiflexion (i.e., foot drop) due to a chronic spinal cord lesion participated in the across-day series of experiments. Thus, participants with SCI were studied only with the across-day experiments. For each participant with SCI, a physiatrist or a neurologist determined each prospective individual’s eligibility for the study. The profiles of participants with SCI are summarized in Table 1. The inclusion criteria were (1) neurologically stable (> 1 year post SCI); (2) medical clearance to participate; (3) ability to ambulate at least 10 m with or without an assistive device (e.g., walker, cane, and crutches); (4) signs of weak ankle dorsiflexion at least unilaterally (i.e., manual muscle strength test score < 5); (5) medically stable at the time of study enrollment. Stable use of anti-spasticity medication (e.g., baclofen, diazepam, tizanidine) was permitted. Exclusion criteria were (1) motoneuron injury; (2) known cardiac condition (e.g., history of myocardial infarction, congestive heart failure, pacemaker use); (3) medically unstable condition; (4) severe cognitive impairment; (5) a history of epileptic seizures; (6) metal implants in the cranium, (7) implanted biomedical device in or above the chest (e.g., a cardiac pacemaker, cochlear implant), (8) no measurable H-reflex or MEP on elicitation, and (9) unable to produce any voluntary TA electromyography (EMG) activity. The inclusion criteria for the participants with no known neurological conditions were no history of neurologic injury or disease and no history of orthopedic injury to the tested side.

**Table 1:** Profiles of study participants with chronic incomplete SCI

Pt. ID	Age (years)	Sex	Cause	SCI level	AIS	Years post SCI	Baclofen
1	52	M	T	C5	D	3	N
2	67	M	NT	T5	D	10	Y
3	56	M	T	C7	D	3	Y
4	35	F	T	C6	D	1.5	Y
5	29	M	T	C7	C	6.5	N
6	39	F	T	C4	C	6	Y
7	45	M	T	C4	D	10	Y
8	48	M	NT	T8	D	6	Y
9	39	F	T	C4	C	4.5	N
10	63	M	T	C4	C	2.5	N
11	47	M	T	C4	C	4	Y
12	42	M	T	C4	D	1.5	Y
13	54	M	T	C6	D	10	N
14	55	F	NT	C6	D	5.5	N
15	57	M	T	C4	D	2	Y
16	58	M	T	C3	D	7	N
17	61	M	T	C5	D	8	Y

Cause: cause of spinal cord damage (*T* trauma, *NT* non-trauma)

The study protocol was approved by the Institutional Review Boards of Helen Hayes Hospital New York State Department of Health and the Medical University of South Carolina, and all participants gave written consent prior to participation.

### General procedure

In the present study, the corticospinal and spinal responses were examined in two separate series of experiments: the within-day protocol and the across-day protocol. With the within-day protocol, the measurements were made at 8:00, 12:00, 16:00, and 20:00 within a 12-h period of the same day. With the across-day protocol, the measurements were made in 4 separate sessions that occurred at the same time across 4 different days, separated by at least one day of rest. Participants were instructed to not refrain from their usual activities or diet but were asked not to introduce anything new or different during or in between testing days. Current medications and dietary and exercise habits were recorded during the screening sessions.

All measurements were made while the participant comfortably sat in a chair with the testing leg fixed in a custom-made apparatus that held the hip, knee, ankle joint angles at approximately 70°, 60°, and − 10° (i.e., 10° of plantarflexion at the ankle), respectively. After EMG recording electrodes were placed over the TA and soleus, and the common peroneal nerve (CPn) stimulating electrodes were placed at the neck of fibula, the maximum voluntary contraction (MVC) level of TA EMG was measured as the mean rectified EMG amplitude. The TA H-reflex–M-wave recruitment curve was then measured using the CPn stimulation while the participant maintained about 15% MVC level of rectified TA EMG activity (in participants with no known neurological injuries) and at rest (in participants with SCI) (see below, Electrical stimulation and EMG recording). Stimulus intensity was increased from the H-reflex or M-wave (whichever occurs at a weaker stimulus) threshold to the maximum H-reflex (*H*_max_) to an intensity just above that needed to elicit the maximum M-wave (*M*_max_) in increments of 1.25–2.5 mA (Kido et al. 2004a, b; Thompson et al. 2006, 2013a; Makihara et al. 2014). Approximately ten different intensities were used to obtain each recruitment curve, and four EMG responses were averaged to measure the H-reflex and M-wave at each intensity. Following the CPn stimulation, the input–output relation of the corticospinal pathway for the TA was obtained by increasing the transcranial magnetic stimulation (TMS) intensity from below the MEP threshold to the level at which the MEP reached its maximum amplitude (MEP_max_). Ten-to-twenty MEPs were then measured at the TMS intensity that originally produced a half-maximum MEP (MEP_h_; typically, 10–20% above threshold) (Knash et al. 2003; Kido Thompson and Stein 2004; Thompson et al. 2011). All MEP measurements were made while the participant voluntarily maintained ≈ 15% MVC level (for participants without SCI) or ≈ 30% MVC level (for participants with SCI) of TA EMG activity. Silent periods after MEP were also measured at the MEP_max_ (SP_max_) and at the MEP_h_ (SP_h_). A typical first measurement session took approximately 45 min, including the time for determining the TMS optimum location and mapping all the stimulating and EMG recording electrode locations. For subsequent sessions, all the measurements were completed within 20 min.

### EMG recordings and electrical stimulation

EMG signals were obtained from the TA and soleus using surface self-adhesive Ag–AgCl electrodes (2.2 × 3.5 cm, Vermed, Inc., Bellows Falls, VT). EMG recording electrodes were placed over the muscle belly for the TA approximately 1/3 the distance between the fibular head and the medial malleolus and about 2 cm below the gastrocnemius in line with the Achilles tendon for the soleus, with their centers ~ 3 cm apart. The signals were amplified and band-pass filtered at 10–1000 Hz (Bortec Biomedical Ltd., Calgary, Canada), and sampled at 3200–5000 Hz. During experimental sessions, EMG signals were also rectified online, averaged every 100 ms, and shown on the feedback screen in front of the participant, so that the participant could monitor the ongoing EMG activity level. For the TA MVC EMG amplitude measurements, the absolute EMG was measured in three trials of ≈ 3-s maximum isometric dorsiflexion effort, separated by at least 1 min of rest in-between.

For the CPn stimulation, the cathode electrode (2.2 × 2.2 cm, Vermed, Inc.) was placed at the neck of fibula and the anode electrode (2.2 × 3.5 cm, Vermed, Inc.) was located at about 2 cm anterior to the cathode. Positions of the stimulating electrode pair were carefully adjusted such that the least amount of stimulus current was required to elicit the M-wave in the TA. CPn stimuli to elicit the M-wave and H-reflex in the TA were 0.5-ms single square pulses delivered from Grass S48 stimulator with SIU-5 stimulation isolation unit and CCU1 constant current unit (Natus Neurology, Warwick, RI). The stimulation was triggered when the participant maintained about 15% MVC level of TA and the resting level (i.e., < 7 μV) of soleus background EMG activity (in participants without SCI) or the resting level (i.e., < 7 μV) of TA and soleus background EMG activity (in participants with SCI) for at least 2 s and at least 5 s had passed since the last stimulus. Note that in participants with SCI, the TA H-reflex–M-wave recruitment curve was measured at rest to reduce the number of trials for which the participant would have to produce and maintain a preset level of voluntary TA activation; this was done to minimize the potential muscle fatigue from affecting MEP measurements.

To avoid session-to-session variability in electrode placement, CPn stimulating, and EMG recording electrode positions were carefully mapped in relation to landmarks on the skin (e.g., scars or moles), and the same investigator prepared the skin and placed the electrodes every session for every participant.

### Transcranial magnetic stimulation

In each experimental session, MEP measurements were made while the participant was seated comfortably in a chair with hip, knee, and ankle joints fixed in a custom-made apparatus, keeping the angles of these joints at approximately 70°, 60°, and − 10°, respectively. Single-pulse monophasic TMS was delivered from MagStim 200^–2^ (Jali Medical Inc., Woburn, MA) via a custom-made, batwing coil with radii of 8 cm; the coil is a modified version of figure-of-8 coil with the outer halves of the two circular coils slightly (25°) bent inward. The coil was held over the scalp such that the induced current flowed in the posterior–anterior direction in the brain. TMS was triggered when the participant had voluntarily maintained ≈ 15% MVC level (in participants without SCI) or ≈ 30% MVC level (for participants with SCI) of TA EMG activity for at least 2 s and at least 5.5 s has passed since the last stimulus. Note that the TA background EMG level differed between neurologically normal participants (≈ 15% MVC) and participants with SCI (≈ 30% MVC), due to their typical differences in ease of voluntary EMG control, MEP size development, and MEP stability at different background EMG levels (Devanne et al. 1997; Garvey et al. 2001). In this study, for background TA activity level for MEP measurements in participants with no injuries, we chose ≈ 15% MVC level, because it is roughly the level of TA activity observed across the swing-phase of walking (Kido et al. 2004a, b). Then, in participants with SCI, in whom higher background EMG (i.e., higher than ≈ 15% MVC level) would help to reduce the TMS intensity to elicit MEPs and help to measure MEPs of more substantial amplitudes (Davey et al. 1999), we aimed to set TA background EMG amplitude close to the level (in mV) generated by individuals without SCI; this led us to using ≈ 30% MVC level for TA background EMG in previous MEP studies in people with weak dorsiflexion due to SCI (Thompson et al. 2011, 2018) and it worked well. Thus, in this study, in participants with SCI, the TA background EMG level was generally aimed at ≈ 30% MVC level.

In the initial session, the optimal TMS location was determined as the location at which the lowest stimulus intensity was required to activate the TA, by moving the coil over the motor areas of the scalp, typically from 0 to 2 cm lateral to and from − 1 to 2 cm posterior to the vertex (corresponding to Cz of the international 10–20 system for EEG). For all subsequent sessions, the same investigator measured and located the participant’s vertex; the same investigator held the TMS coil to minimize the session-to-session variability in a given participant’s MEP measurements; and TMS was applied at the optimal location (e.g., 1 cm lateral and 1 cm posterior to the vertex) determined in the initial session. At the optimal location, the input–output curve was measured by increasing the TMS intensity, expressed as a percentage of the maximum stimulator output (MSO), in steps of 5% from below the MEP threshold until the MEP reached its plateau (MEP_max_). A typical TMS intensity range used for the input–output curve measurement was 40–70% MSO for participants without SCI, and the range was the same for participants with SCI in whom MEP_max_ could be obtained. Four MEPs were collected at each stimulus level, and the intensity eliciting a half-maximum MEP (MEP_h_) was determined as the closest to the estimated parameter *h*, after fitting the data with a sigmoid curve of the form:

$$y = b + \left( {m{-}b} \right)/\left( { \, 1 + \exp \left( {h{-}x} \right)/w} \right),$$

where *y* is the MEP, *b* is the background activity in the absence of an MEP, *x* is % of the TMS maximum stimulator output, *m* is the maximum MEP, *h* is the stimulus level producing a half-maximum MEP, and *w* is a measure of the width of the curve (Devanne et al. 1997; Knash et al. 2003; Kido Thompson and Stein 2004; Thompson et al. 2011). We collected 10–20 MEPs evoked at this TMS intensity for a MEP_h_ (typically 10–20% above estimated threshold [as the minimum TMS intensity required to induce MEPs of > 0.1 mV in 50% of the trials (Rossini et al. 1994)].

Note that in 9 participants with SCI the full MEP recruitment curve could not be obtained and the MEP_max_ could not be determined; this was because the maximum stimulator output of TMS was not sufficient to attain the MEP_max_ or the higher TMS intensity needed for MEP_max_ measurement could not be tolerated. In those individual, MEP_h_ measurements were made at the TMS intensity of 10–20% above the estimated threshold, based on the slope of the available MEP recruitment curve data.

### Data analysis

In this study, we measured the TA MVC, *M*_max_, *H*_max_, MEP_max_, MEP_h_, SP_max_ (SP duration at the MEP_max_) and SP_h_ (SP duration at the MEP_h_). For the evoked potentials (i.e., *M*_max_, *H*_max_, MEP_max_, MEP_h_) the mean value was calculated as the peak-to-peak amplitude in each session in each participant. For the MVC, the mean EMG amplitude (in absolute value) over the period of MVC effort was calculated in each of the three MVC attempts, and then the average of the three was reported as the TA MVC. Silent periods (SP_max_ and SP_h_) were measured as the period from the end of the MEP to the recovery of background EMG activity in 50% of responses (Garvey et al. 2001; Knash et al. 2003; Kido Thompson and Stein 2004; Thompson et al. 2011). End of MEP was determined, on the full wave rectified EMG signal, as the time when the MEP EMG burst diminished and fell below the pre-stimulus (i.e., background) EMG level. Note that in 4 participants with SCI, SP_h_ could not be determine as the recovery of background EMG activity was not observed.

### Statistical analysis

For each of the seven above-mentioned EMG measures (i.e., MVC, *M*_max_, *H*_max_, MEP_max_, MEP_h_, SP_max_, and SP_h_) we perform the following analyses. First, for the within-day dataset and across-day dataset in participants without SCI, and for the across-day dataset in participants with SCI, we calculated the standard error of measurement (SEM). We also calculated the coefficient of variation (CV) per individual. Then, the CV values were compared between the protocols (within-day and across-day) and cohorts (participants with SCI and participants without SCI) by *t* test. For each participant and for each measure, the mean absolute deviation of four measurements was also calculated. We computed the intraclass correlation (ICC) assuming random participants and random sessions (ICC(2, 1)) (McGraw and Wong 1996). ICC values range from 0 to 1, with 1 denoting perfect repeatability across sessions. ICC values of greater than 0.6 are considered supportive of reasonably high repeatability (McGraw and Wong 1996). To quantify the mean variance across the four sessions and to test for significant predictors of the EMG measures, for the data from participants with no neurological injuries, linear mixed models (LMM) that considered the fixed effects of PROTOCOL (i.e., within-day vs. across-day) and SESSION (i.e., four measurement time points either within-day or across-days) with participant as the random effect (intercept) were used for analyses. These models assumed random intercepts and slopes for each participant to account for the longitudinal (i.e., repeated measures) design. For the data from participants with SCI, a similar mixed effects model was employed to assess the effect of SESSION with participant level random intercepts and slopes and an overall fixed slope effect.

The R language (version 4.3.1, R Core Team 2023) was used for all statistical analyses. We used the R packages *lme4* for mixed effects modeling (Bates et al. 2015) and *irr* for ICC estimates (Gamer et al. 2019). Power calculations were performed using the *simr* package (Green and MacLeod 2016) in R.

## Results

### Reliability and stability of repeated EMG measurements

Prior to assessing the presence and extent of physiological variability in EMG measures, we assessed the reliability and variability of repeated measurements by calculating SEM and CV. Mean and SEM values for *M*_max_, *H*_max_, MEP_max_, MEP_h_, SP_max_, SP_h_, and MVC are summarized in Table 2. For CV in participants without SCI, the values were compared between the within-day and across-day protocol groups. CVs were below 20% on average (except for *M*_max_ for which CVs were even smaller) and did not differ between the protocol groups; *M*_max_: 6.9 ± 2.6 (mean ± SD)% vs. 5.8 ± 3.1% (*p* = 0.37 by two-tailed *t* test); *H*_max_: 17.2 ± 9.5% vs. 14.6 ± 6.9% (*p* = 0.51); MEP_max_: 10.5 ± 5.8% vs. 15.1 ± 6.2% (*p* = 0.09); MEP_h_: 14.6 ± 7.1% vs. 18.7 ± 10.9% (*p* = 0.30); SP_max_: 11.7 ± 9.5% vs. 14.6 ± 8.5% (*p* = 0.48); SP_h_: 16.7 ± 9.0% vs. 11.6 ± 5.3% (*p* = 0.15); and MVC: 13.0 ± 6.8% vs. 9.5 ± 7.2% (*p* = 0.27). CVs in participants with SCI were 10.3 ± 3.0% for *M*_max_, 21.3 ± 12.8% for *H*_max_, 19.1 ± 5.5% for MEP_max_, 8.9 ± 4.1% for MEP_h_, 8.8 ± 4.5% for SP_max_, 11.8 ± 5.6% for SP_h_, and 11.7 ± 7.5% for MVC. When these CVs were compared to the across-day group of participants without SCI by *t* test, *p* = 0.002, 0.41, 0.72, 0.005, 0.32, 0.84, and 0.87 for these seven measures, respectively.

**Table 2:** Mean values of the TA *M*_max_, *H*_max_, MEP_max_, MEP_h_, SP_max_, SP_h_, and MVC in participants with no known neurological conditions and participants with SCI

	Participants without SCI				Participants with SCI	
	Within-day (*N* = 9)		Across-day (*N* = 13)		Across-day (*N* = 17^a^)	
	Mean	SEM	Mean	SEM	Mean	SEM
*M*_max_ (mV)	4.4 ± 1.2	0.33	4.6 ± 2.0	0.26	2.6 ± 1.2	0.26
*H*_max_ (mV)	0.5 ± 0.2	0.10	0.5 ± 0.2	0.08	0.5 ± 0.8	0.11
MEP_max_(mV)	1.2 ± 0.4	0.13	1.3 ± 0.6	0.20	0.5 ± 0.4	0.11
MEP_h_ (mV)	0.6 ± 0.3	0.09	0.7 ± 0.3	0.19	0.4 ± 0.2	0.04
SP_max_ (ms)	102 ± 23	14.1	104 ± 24	19.0	86 ± 22	10.4
SP_h_ (ms)	61 ± 25	11.4	62 ± 27	8.4	79 ± 28	9.5
MVC (µV)	250 ± 57	37.1	325 ± 83	33.8	118 ± 51	15.0

^a^All values were averaged per participant (across 4 measurements) first, and then the group mean (± SD) values were calculated. In participants with SCI, the sample size was 17 except for MEP_max_ (*N* = 8), SP_max_ (*N* = 8), and SP_h_ (*N* = 13)

**Fig. 1:**
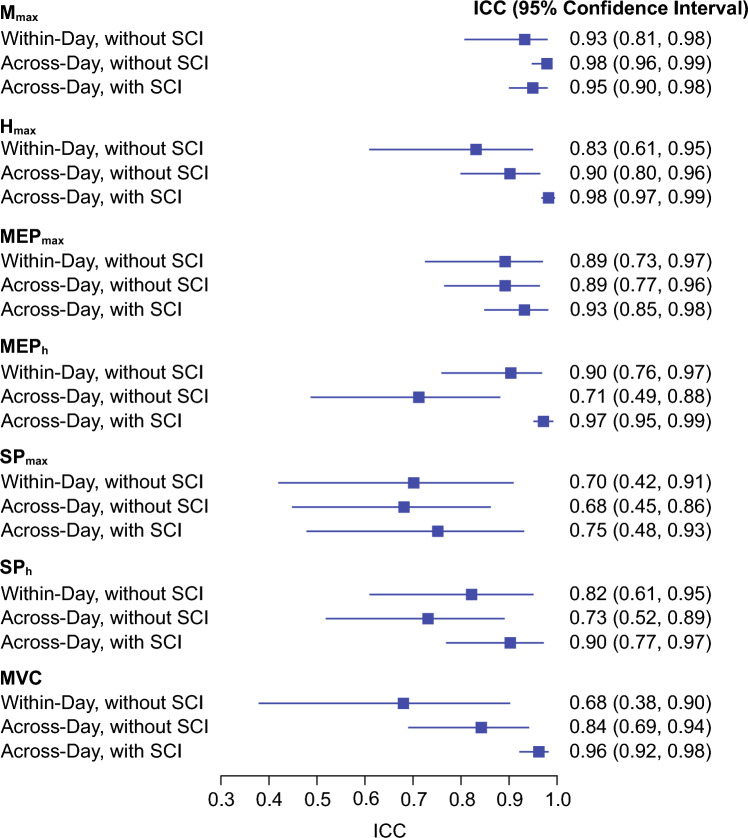
Forest plot of intraclass correlation coefficient (ICC) values (with 95% confidence intervals)

We also assessed the reproducibility of EMG measurements by calculating the ICC. For the within-day protocol in participants with no known neurological injuries, ICCs were 0.83, 0.93, 0.89, 0.68, 0.82, 0.70, and 0.90 for *M*_max_, *H*_max_, MEP_max_, MEP_h_, SP_max_, SP_h_, and MVC, respectively. For the across-day protocol in participants with no known neurological injuries, ICCs were 0.98, 0.90, 0.89, 0.71, 0.68, 0.73, and 0.84 for *M*_max_, *H*_max_, MEP_max_, MEP_h_, SP_max_, SP_h_, and MVC, respectively. For the across-day protocol in participants with SCI, ICCs were 0.95, 0.98, 0.93, 0.97, 0.75, 0.90, and 0.96 for *M*_max_, *H*_max_, MEP_max_, MEP_h_, SP_max_, SP_h_, and MVC, respectively (see Fig. 1).

TA background EMG for these measurements were, on average, 43 μV (with SEM of 3.0 μV) for the within-day protocol group and 47 μV (with SEM of 3.5 μV) for the across-day protocol group of participants without SCI, corresponding to 17% MVC and 15% MVC, respectively. For the participants with SCI, TA background EMG for MEP measures were 35 μV (with SEM of 2.9 μV) on average, corresponding to 30% MVC. The ICCs calculated for TA background EMG for these measurements were all > 0.95 (not shown) for the within- and across-day protocols in the non-SCI group and for the across-day protocol in the SCI group. Thus, we can reasonably assume that TA background EMG was maintained stable across multiple sessions for each protocol and group in this study.

### Within-day and across-day variability in the excitability of corticospinal and spinal pathways in people without neurological injuries

**Fig. 2:**
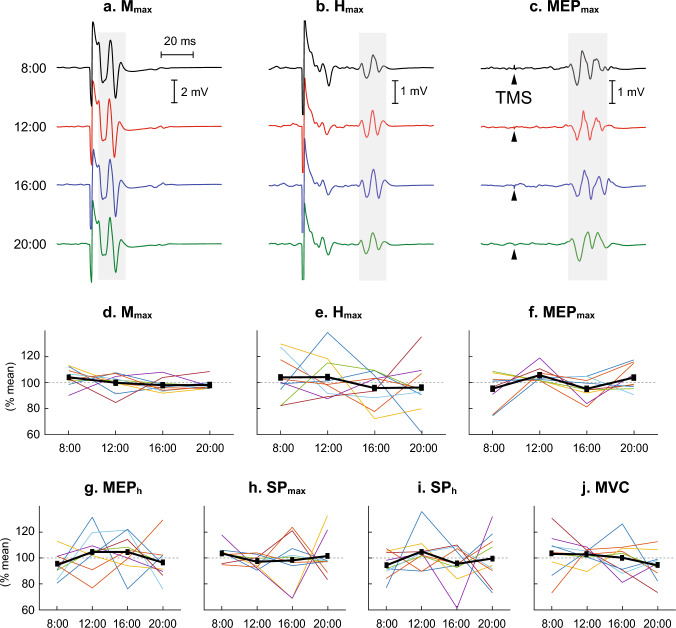
Measures of corticospinal and spinal excitability for the tibialis anterior (TA) repeated at 4 different times during a single day in participants with no known neurological disorders. **a**–**c** Peristimulus TA EMG sweeps for the *M*_max_, *H*_max_ and MEP_max_ from a single participant. For each sweep ≈ 4 responses are averaged together. The grey/shaded area indicates the time window for which each variable was measured. **d**–**j** The mean value across all participants (thick black line) and individual participant values (each line for each participant) for *M*_max_, *H*_max_, MEP_max_, MEP_h_, SP_max_, SP_h_, and MVC (*N* = 9). All values are expressed as %mean value

**Fig. 3:**
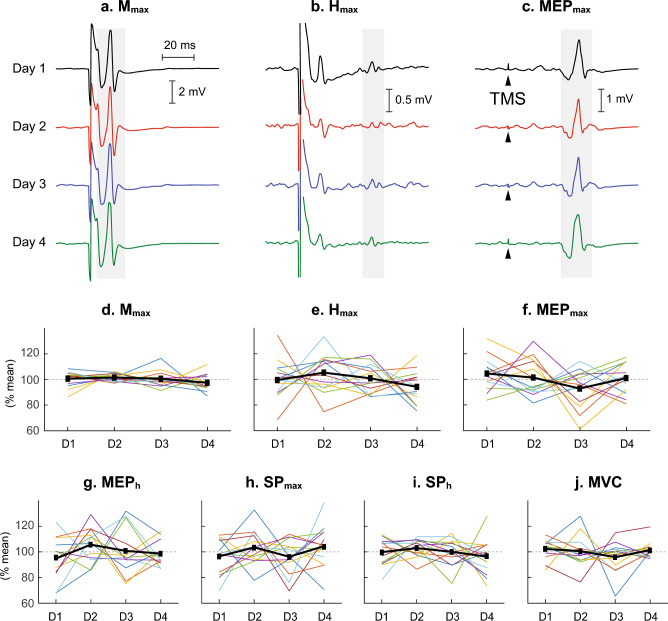
Measures of corticospinal and spinal excitability for the tibialis anterior (TA) repeated over four different days at approximately the same time of day in participants with no known neurological disorders. **a**–**c** Peristimulus TA EMG sweeps for the *M*_max_, *H*_max_ and MEP_max_ from a single participant. For each sweep ≈ 4 responses are averaged together. The grey/shaded area indicates the time window for which each variable was measured. **d**–**j** The mean value across all participants (thick black line) and individual participant values (each line for each participant) for *M*_max_, *H*_max_, MEP_max_, MEP_h_, SP_max_, SP_h_, and MVC (*N* = 13). All values are expressed as %mean value

TA *M*_max_, *H*_max_, MEP_max_, MEP_h_, SP_max_, SP_h_, and MVC in participants without SCI, across four measurement time points within-day and across 4 days, are expressed in %mean (i.e., the mean of the four measurements) and presented in Figs. 2, 3. As seen here there are no clear systematic (e.g., linear, or monotonic) increases or decreases across 4 sessions in the measures obtained in this study in participants without neurological disease or injury.

To examine if within-day or across-day protocol affected the EMG measures over the course of four repeated measurements, we fitted a LMM to predict change in those EMG measures with PROTOCOL and SESSION. For *M*_max_, the model’s total explanatory power was very large (conditional *R*^2^ = 0.96), and the part related to the fixed effects alone was very small (marginal *R*^2^ = 0.002). The main effect of SESSION (*p* = 0.48), PROTOCOL (*p* = 0.24), or PROTOCOL × SESSION interaction (*p* = 0.32) was not significant. For *H*_max_, conditional *R*^2^ = 0.90 and marginal *R*^2^ = 0.007; the effect of SESSION (*p* = 0.22), PROTOCOL (*p* = 0.27), or PROTOCOL × SESSION interaction (*p* = 0.82) was not significant. For MEP_max_, conditional *R*^2^ = 0.87 and marginal *R*^2^ = 0.002; the effect of SESSION (*p* = 0.44), PROTOCOL (*p* = 0.99), or PROTOCOL × SESSION interaction (*p* = 0.47) was not significant. For MEP_h_, conditional *R*^2^ = 0.71 and marginal *R*^2^ = 0.036; the effect of SESSION (*p* = 0.87), PROTOCOL (*p* = 0.11), or PROTOCOL × SESSION interaction (*p* = 0.87) was not significant. For SP_max_, conditional *R*^2^ = 0.68 and marginal *R*^2^ = 0.001; the effect of SESSION (*p* = 0.65), PROTOCOL (*p* = 0.90), or PROTOCOL × SESSION interaction (*p* = 0.67) was not significant. For SP_h_, conditional *R*^2^ = 0.68 and marginal *R*^2^ = 0.001; the effect of SESSION (*p* = 0.11), PROTOCOL (*p* = 0.47), or PROTOCOL × SESSION interaction (*p* = 0.40) was not significant. For MVC, conditional *R*^2^ = 0.83 and marginal *R*^2^ = 0.046; the effect of SESSION (*p* = 0.35), PROTOCOL (*p* = 0.07), or PROTOCOL × SESSION interaction (*p* = 0.72) was not significant.

To estimate the extent of within-day and across-day variability, the mean (absolute) deviation across 4 sessions was calculated for each measure, and the values are summarized in Table 3.

**Table 3:** Mean absolute deviations across four measurements for the TA *M*_max_, *H*_max_, MEP_max_, MEP_h_, SP_max_, SP_h_, and MVC in participants with and without chronic incomplete SCI

	Participants without SCI				Participants with SCI	
	Within-day (*N* = 9)		Across-day (*N* = 13)		Across-day (*N* = 17^a^)	
	% mean	Raw value	% mean	Raw value	% mean	Raw value
*M*_max_	5.1 ± 1.8	0.23 ± 0.11 mV	4.5 ± 2.4	0.18 ± 0.08 mV	7.4 ± 2.6	0.18 ± 0.08 mV
*H*_max_	12.9 ± 7.3	0.07 ± 0.04 mV	11.2 ± 5.3	0.05 ± 0.03 mV	12.8 ± 8.0	0.05 ± 0.06 mV
MEP_max_	7.6 ± 4.2	0.08 ± 0.05 mV	11.2 ± 4.6	0.14 ± 0.06 mV	11.7 ± 4.8	0.06 ± 0.04 mV
MEP_h_	10.9 ± 5.6	0.06 ± 0.03 mV	12.2 ± 5.4	0.09 ± 0.08 mV	6.3 ± 2.6	0.02 ± 0.02 mV
SP_max_	8.8 ± 6.9	8.4 ± 6.2 ms	11.2 ± 6.7	12.0 ± 9.4 ms	7.2 ± 4.0	7.1 ± 3.2 ms
SP_h_	12.6 ± 6.2	7.4 ± 3.9 ms	10.9 ± 6.8	5.5 ± 3.3 ms	8.3 ± 3.8	6.4 ± 3.5 ms
MVC	9.8 ± 5.2	24 ± 15 µV	7.1 ± 5.3	21 ± 13 µV	7.3 ± 4.7	8 ± 7 µV

^a^All values are presented as mean ± SD. In participants with SCI, the sample size was 17 except for MEP_max_ (*N* = 8), SP_max_ (*N* = 8), and SP_h_ (*N* = 13)

### Across-day variability in the excitability of corticospinal and spinal pathways in people with SCI

**Fig. 4:**
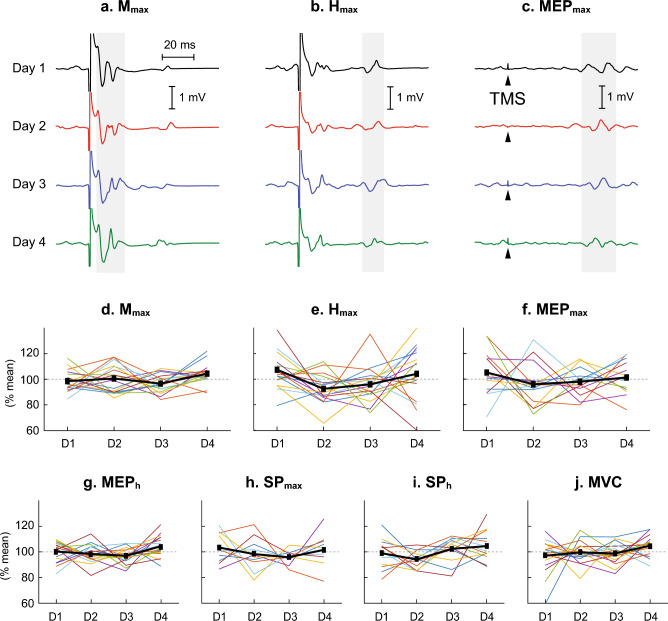
Measures of corticospinal and spinal excitability for the tibialis anterior (TA) repeated over 4 different days at approximately the same time of day in participants with chronic incomplete SCI. **a**–**c** Peristimulus TA EMG sweeps for the *M*_max_, *H*_max_ and MEP_max_ from a single participant. For each sweep ≈ 4 responses are averaged together. The grey/shaded area indicates the time window for which each variable was measured. **d**–**j** The mean value across all participants (thick black line) and individual participant values (each line for each participant) for *M*_max_ (*N* = 17), *H*_max_ (*N* = 17), MEP_max_ (*N* = 8), MEP_h_ (*N* = 17), SP_max_ (*N* = 8), SP_h_ (*N* = 13), and MVC (*N* = 17). All values are expressed as % mean value

TA *M*_max_, *H*_max_, MEP_max_, MEP_h_, SP_max_, SP_h_, and MVC in participants with SCI, across 4 days of measurements are expressed in % mean and presented in Fig. 4. Besides some inter-individual variabilities, there are no obvious systematic increases or decreases across 4 sessions in the measures obtained. Values of the mean deviation across 4 sessions are summarized in Table 3.

To examine the trend of variation in the EMG measures over the course of 4 repeated measurements, we fitted a LMM to predict change in those EMG measures with SESSION. For *M*_max_, the model's total explanatory power was very large (conditional *R*^2^ = 0.95), and the part related to the fixed effects alone was small (marginal *R*^2^ = 0.001). The effect of SESSION (*p* = 0.41) was not significant. For *H*_max_, conditional *R*^2^ = 0.99 and marginal *R*^2^ = 0.000; the effect of SESSION (*p* = 0.39) was not significant. For MEP_max_, conditional *R*^2^ = 0.92 and marginal *R*^2^ = 0.000; the effect of SESSION (*p* = 0.78) was not significant. For MEP_h_, conditional *R*^2^ = 0.98 and marginal *R*^2^ = 0.001; the effect of SESSION (*p* = 0.21) was not significant. For SP_max_, conditional *R*^2^ = 0.83 and marginal *R*^2^ = 0.002; the effect of SESSION (*p* = 0.56) was not significant. For SP_h_, conditional *R*^2^ = 0.93 and marginal *R*^2^ = 0.010; the effect of SESSION (*p* = 0.09) was not significant. For MVC, conditional *R*^2^ = 0.93 and marginal *R*^2^ = 0.002; the effect of SESSION (*p* = 0.21) was not significant.

## Discussion

This study aimed to examine the variability and stability of TA corticospinal excitability across repeated measurement sessions in individuals with and without corticospinal lesions. For all 7 EMG measures, which would help to evaluate corticospinal and spinal reflex excitability for TA, calculation of SEM and assessment of CV and ICC suggested generally high reliability and low variability of those measures across 4 repeated experimental sessions. Through LMM analyses, we found no systematic fluctuation in those EMG measures over 4 days or over 12 h within the same day in participants without SCI, and across 4 days in participants with chronic incomplete SCI. Below we discuss implications of those repeatability and variability assessments and the absence of systematic day-to-day or diurnal variability in the measures of TA corticospinal and spinal excitability for the existing and future clinical research studies that investigate corticospinal plasticity.

### Repeatability and variability in corticospinal or spinal evoked responses across multiple measurements

In the present study, the mean absolute deviations across four measurements remained in a range of 5–13% in all the corticospinal and spinal excitability measures. In participants without SCI, CVs, which indicate the extent of variability in relation to the mean of the multiple measurements, were 6–19% across 7 different EMG measures. These percentages are in support of limited variability in these measures when the measurements are repeated. The fact that CVs did not differ between the within-day and across-day protocols suggest that the extent of potential diurnal variability in corticospinal and spinal reflex excitability (if present) remains within a day-to-day (or session-to-session) variability range. ICC values, which indicate the repeatability of the measurements, were also high (i.e., ≥ 0.68, often over 0.90) for the present measures, suggesting excellent measurement-to-measurement reliability/reproducibility (Koo and Li 2016) can be achieved in participants without SCI. The present values are in agreement with a couple of existing studies; Palmieri et al. (2002) reported ICC values of 0.78–0.997 for the TA *H*_max_, *M*_max_, and *H*_max_/*M*_max_ ratio, and Tallent et al. (2012) reported ICC values of 0.81–0.94 for TA MEPs across 3 days and ICC values of > 0.66 for TA H-reflex across 3 days during isometric and dynamic muscle contraction. Together with our history of successful long-term repeated (i.e., 30–40 sessions) EMG evoked response measurement studies (Thompson et al. 2009, 2013b, 2018, 2022; Makihara et al. 2014; Mrachacz-Kersting et al. 2019; Thompson and Wolpaw 2019, 2021) and existing studies by the other groups, the present CV and ICC results support that well-maintained experimental setups and stable administration of experimental procedures would lead to low variability and high repeatability of EMG measures across multiple measurement sessions, which in turn would help us assess the presence and extent of physiological changes and plasticity.

In addition, based on the limited measurement-to-measurement variabilities observed in the present study, we may assume that in people with no known neurological conditions, the measures of TA corticospinal and spinal excitability may vary but would remain generally within a ± 15% range across multiple measurement sessions when the experimental conditions are kept the same. Still, the variability in corticospinal and spinal excitability measures would likely be affected by specific methodologies employed in each study; those include (but not limited to) sample size, number of responses averaged together, participants' characteristics, experimental setup (e.g., background EMG feedback vs. force feedback, or active EMG vs. no active EMG), EMG amplifier that affects the signal-to-noise ratio, and so on. Thus, the assessment of TA corticospinal excitability, for example, in response to a neuromodulatory intervention, resulting in small (e.g., less than 15%) changes in EMG evoked response measures, may need to be considered carefully through rigorous statistical and qualitative analyses.

In participants with SCI, CVs were 9–21% across different EMG measures. Within a relatively small range, the CV for *M*_max_ was larger and the CV for MEP_h_ was smaller than that in participants without SCI. Besides the difference in background EMG setting used during these measurements potentially affecting the measurement-to-measurement variability (note that background EMG concerns are discussed further in the section *Methodological considerations, limitations, and clinical implications*), these differences in CVs may be partly related to the raw amplitude of those measures in individuals with SCI. As shown in Tables 2 and 3, TA *M*_max_ tends to be smaller in mV in participants with SCI than participant without while their SEMs and mean deviations are comparable to those in participants without SCI; these would result in larger CVs in participants with SCI. For MEP_h_, its smaller CV may be because the entire MEP recruitment curve tends to reside within a narrower range in participants with SCI; that is, because not only MEP_h_ but also MEP_max_ is small, the range within which MEP_h_ can fluctuate would naturally be limited. As a result, the CV for MEP_h_ would be small (and so as SEM and mean deviation). It should be noted that despite its statistical difference from non-SCI, the CV for *M*_max_ in participant with SCI is 10% (and mean deviation is 7%) on average, and thus, the physiological significance of such a limited extent of variability is not clear. For the MEP_h_, it is also not clear if the CV of 9% (and mean deviation of 6%) implies anything positive or negative in terms of how the corticospinal pathway functions in this population. In participants with SCI, ICC values were all high (i.e., 0.90–0.98 except for 0.75 in SP_max_), suggesting excellent measurement-to-measurement reproducibility (Koo and Li 2016) for the TA EMG measures in people with SCI.

### Absence of the effects of repeated measurements either within-day or across days

In individuals with no known neurological conditions, the present LMM analyses yielded no effects of SESSION, indicating that there were no systematic fluctuations across four time points of measurements in any of the EMG measures that reflect corticospinal or spinal reflex excitability. This implies two things. First, repeating the same measurements four times with 3+ h intervals (i.e., within-day protocol) or > 23-h intervals do not produce systematic effects on the EMG measures that were obtained in this study. Second, the corticospinal or spinal reflex excitability for the TA would not vary systematically within or across days (over four measurement sessions).

In individuals with chronic incomplete SCI, LMM analyses on the session-to-session across-day variability in the EMG measures of TA corticospinal and spinal excitability exhibited no systematic changes across measurements, similarly to the findings in individuals with no known neurological conditions. This further indicates that in individuals with SCI who are medically stable and in good health, partial damage to the spinal cord does not necessarily create some unknown cumulative effects of four repeated measurements with > 23-h intervals.

Note that the extent of session-to-session variability appeared less for *M*_max_ than other measures in individuals with no known neurological conditions (Figs. 2 and 3 and Table 3). This was expected, because *M*_max_ is from stimulating all motor axons (Pierrot-Deseilligny and Burke 2012), its values would not be affected by the motoneuron pool excitability or synaptic input converging onto motoneurons. Therefore, the stability of *M*_max_ supports the reproducibility of experimental setup across multiple experiments. In contrast, the H-reflex and MEP are from transsynaptic excitation of motoneurons, and thus, would reflect the above factors and presynaptic mechanisms that influence the efficacy of synaptic input onto motoneurons (Capaday and Stein 1987; Stein 1995). In the present study, because the experiments were set up to keep the task, posture, and ongoing EMG activity consistent across different MEP and H-reflex measurements, the session-to-session variability in motoneuron pool excitability and task- and posture-dependent pre- and postsynaptic inhibition was presumably minimum. Interpretation of the resulting variability could, therefore, be about what was measured and when. We found no consistent changes in magnitude and/or pattern, for the measures of corticospinal excitability (i.e., MEP_max_ and MEP_h_), spinal reflex excitability (i.e., *H*_max_), and cortical (at least partly) inhibition (i.e., SP_max_ and SP_h_), across 4 days (in individuals with or without SCI) or over 12 h period (in individuals without SCI) for the ankle dorsiflexor TA; and none varied more (or less) or systematically when the same measurements are simply repeated multiple times.

### Absence of clear diurnal variation in the corticospinal and spinal excitability for the TA

In this study, we did not observe any diurnal variation in the corticospinal or spinal reflex excitability of the TA in individuals with no known neurological conditions. To our knowledge, this is the first study to look at diurnal variation in the TA. Previous studies have shown diurnal variation exists in the corticospinal excitability (Tamm et al. 2009) and H-reflex amplitude (Lagerquist et al. 2006) of other muscles (e.g., the triceps surae). This suggests that diurnal variation in the corticospinal and spinal excitability is not a universal phenomenon across leg muscles. This was somewhat unexpected since diurnal rhythms exist in multiple physiological processes throughout the human body, including body temperature (Aschoff 1983; Refinetti and Menaker 1992), blood pressure and heart rate (Millar-Craig et al. 1978; Weber et al. 1984; Degaute et al. 1991), hormonal regulation (Bailey and Heitkemper 2001), sleep/wake cycle (Santhi et al. 2016), and torque/force production (Freivalds et al. 1983; Gauthier et al. 1996, 2001; Martin et al. 1999; Guette et al. 2005b; Lagerquist et al. 2006; Sedliak et al. 2008; Andrews et al. 2010; Hodge et al. 2019; Douglas et al. 2021). The effect of diurnal rhythm on these measures can further be influenced by chronotype (Tamm et al. 2009), gender (Duffy et al. 2011; Santhi et al. 2016), and age (Silva et al. 2021). In particular, voluntary maximal and submaximal isometric force production has been reported to increase throughout the day in several different muscles, such as triceps surae (Castaingts et al. 2004; Guette et al. 2005a, 2006; Lagerquist et al. 2006), quadriceps femoris (Callard et al. 2000; Guette et al. 2005b; Sedliak et al. 2008), biceps brachii (Freivalds et al. 1983; Gauthier et al. 1997, 2001), and adductor pollicis (Martin et al. 1999). At the same time, the extent of diurnal variation in EMG activity during isometric force production could differ even among synergist muscles (Tamm et al. 2009). In a recent review, Douglas et al. (2021) suggested that time of day difference in muscle strength could be a circadian rhythm linked to intrinsic muscle properties such as myosin type and calcium and/or kinase regulation that would affect contractile proteins (Zhi 2005; Andrews et al. 2010; Schroder et al. 2015; Perrin et al. 2018; Hodge et al. 2019; Altıntaş et al. 2020; Swist et al. 2020), rather than neural activation and drive or extrinsic factors such as temperature or wakefulness (Gauthier et al. 1996; Martin et al. 1999; Nicolas et al. 2008; Sedliak et al. 2008; Gueldich et al. 2017). The present study's observations are in line with this view; EMG activity during MVC and the corticospinal and spinal excitability did not exhibit systematic changes throughout the day (i.e., not beyond random variations), leaving the intrinsic muscle property change as a possible mechanism of force production increase throughout the day.

This brings us to the question of why and how diurnal variations in the corticospinal and spinal excitability appear differently between the soleus/triceps surae (Lagerquist et al. 2006; Tamm et al. 2009) and TA (i.e., present study). In the soleus, diurnal rhythms exist both for the corticospinal and spinal excitability (Wolpaw et al. 1984; Chen and Wolpaw 1994; Carp et al. 2006a; Lagerquist et al. 2006; Tamm et al. 2009). In rats, Chen et al. (2002) showed that transection of the corticospinal tract (CST), not lateral column or dorsal column, results in reduction of rhythm amplitude and phase, implying that the soleus H-reflex diurnal rhythm depends on the CST activity that reflects descending drive from sensory and motor-related areas of the cortex. In humans, diurnal rhythms in the soleus corticospinal excitability are observed with chronotypes (Tamm et al. 2009), i.e., the corticospinal excitability is high in the AM in the morning people and high in the PM in the evening people. In parallel, the soleus H-reflex excitability increases throughout the day, regardless of chronotype (Lagerquist et al. 2006; Tamm et al. 2009). These observations are different from the present ones, which showed no systematic increases or decreases in TA MVC, H-reflex, and MEP measures made over a course of 12 h within-day. The difference in the strength of cortical or corticospinal drive may partially explain this TA − soleus difference. Bawa et al. (2002) showed the existence of corticomotoneuronal connections for both the soleus and TA, with more prominent connection for the TA compared to the soleus. Differing strengths in corticospinal drive between these two muscles have also been demonstrated in other studies (Advani and Ashby 1990; Brouwer and Ashby 1992). Thus, it is theoretically possible that in the muscle that is largely controlled by abundant corticospinal drive, relatively small diurnal variations in the drive may be obscured by its random variations and not be readily detectable in EMG measures.

### Methodological considerations, limitations, and clinical implications

In the present study, we used the custom-made batwing coil, which was originally designed to stimulate the leg motor areas of the cortex more effectively than the standard figure-of-8 coil and more focal than the double-cone coil (although the produced electric field might not penetrate deep into the lower leg motor area as effectively as the double-cone coil). While the batwing coil has been used successfully in a number of studies (Barthelemy et al. 2011; Needle et al. 2013; Palmer et al. 2016, 2017a, b; Cai et al. 2012), since the created electric fields would differ between different coil designs, whether and to what extent the current findings with the batwing coil may be replicated with the double-cone coil stimulation are yet to be determined.

In the present study, the TA background EMG levels differed between neurologically normal participants and participants with SCI for *M*_max_ and *H*_max_ measurements (≈ 15% MVC level in non-SCI vs. at rest in SCI) and for MEP measurements (≈ 15% MVC level in non-SCI vs. ≈ 30% MVC level in SCI). Such methodological limitations warrant further consideration in interpreting the data. In this study, we prioritized on measuring MEPs that reflect corticospinal excitability while background EMG was actively controlled so that the motoneuron pool’s excitability could be controlled during the measurements. (Note that eliciting MEPs at rest is possible, but at rest, we could never be certain of the subthreshold level of motoneuron pool’s excitability, which would affect MEPs.) To consider potential concurrent systematic fluctuations in the excitability of corticospinal and spinal reflex pathways, ideally EMG measures to peripheral nerve stimulation should also be made during the same level of background EMG activation as MEP elicitation. However, since it was difficult for many of the current study participants with chronic incomplete SCI (with weak dorsiflexion) to go through many trials with the controlled amount of active background EMG, we elected to drop the H-reflex measurement with active EMG in this group. Admittedly, this resulted in measuring H-reflexes with uncontrolled variability in the subthreshold level of motoneuron pool’s excitability, and thus, the present H-reflex measurements in SCI are not immediately relatable to the variability in MEP amplitude across repeated measurement sessions. *M*_max_, on the other hand, would still serve as an estimate of the response of the entire motoneuron pool (that can be recorded with the specific EMG electrode configuration that is used); and as long as the posture, skin preparation, and electrode positions are consistent, *M*_max_ should be comparable between the measurements and could help to explain potential variations in MEP amplitude. Indeed, the present data (i.e., low CVs and high ICCs) support our confidence in within-participant measurement repeatability, for a give participant and for a given protocol/participant group. That being said, we are aware that the SCI vs. non-SCI comparisons (i.e., comparisons of CVs) are not ideal. Difference in background EMG activity levels could have affected the assessment of session-to-session variability in MEP and H-reflex measures. To our knowledge, whether and to what extent different levels of active background EMG may affect session-to-session variability in MEP measures is currently unknown. Despite such uncertainties, the present study found the session-to-session variability of these measures within a quite reasonable range in participants with SCI.

In this study, we examined the measurement-to-measurement variability in the TA corticospinal and spinal excitability measures in individuals without known neurological conditions across 4 time points within the same day and across 4 days (at the same time of day). By examining the extent and pattern of variability in two different timelines, we hoped to differentiate any potential effect of repeated measurements from a potential diurnal effect on corticospinal and spinal excitability measures if one existed at all. Here we observed no clear effect of repeated measures either over 12 h or across days, with the magnitude of variations being similar within and across days. Because we performed these examinations only in individuals without injuries, some may wonder why we did not do the same in individuals with SCI. It is important to note that in individuals with chronic SCI, examining whether and to what extent the diurnal variation may exist would most likely be meaningless. Many of those individuals are on chronic daily medication and medication effects would not be the same throughout day. Thus, estimating (and accounting for) changes in medication effectiveness throughout day for each individual and across different individuals would not be possible in reality. Since this study did not find diurnal variation in the corticospinal and spinal excitability for TA of the intact CNS, an implication of this for neurorehabilitation is that there is no specific time of day that is neurophysiological optimum for rehabilitation therapy involving TA dysfunction (e.g., therapy aiming to alleviate foot drop). What might be more important in therapy administration is to maintain the intervention/training schedule in relation to daily chronic medication regimens.

Variability will always exist in human neurophysiology and thus in experimental measures. Therefore, knowing what a normal range of variability in a given measure (including the variability introduced by human experimenters) can help to detect neurophysiological changes that are beyond random variations and attributable to a therapeutic protocol or intervention. What this study provides are some reference values as to how much the TA corticospinal and spinal excitability measures may vary in people of stable health with and without chronic incomplete SCI.

## Conclusion

In this study, we examined whether session-to-session variability existed in corticospinal and spinal excitability for the ankle dorsiflexor TA in people with and without incomplete SCI. We found no clear diurnal variation in the measures of spinal and corticospinal excitability of the TA in individuals with no known neurological conditions, and no systematic changes in any experimental measures of spinal and corticospinal excitability across four measurement days in individuals with or without SCI. This study demonstrates that it is possible to repeatedly measure the corticospinal and spinal evoked responses for the TA with stability in people of stable health with or without chronic SCI. This supports the utility of corticospinal and spinal excitability measures in mechanistic investigation of neuromodulation interventions. While systematic variation of corticospinal excitability was not found for the TA, considering complex diurnal variations in multiple human physiological processes and possible effects of daily scheduled medication, the consistency in the time of day to administer an intervention remains essential for scientific integrity and therapeutic efficacy in long-term rehabilitation studies in people with SCI and other neuromuscular disorders.

## Data Availability

Study data are available upon request.
